# Enhancement of Menaquinone-7 production in *Bacillus subtilis* by optimizing the medium components through response surface methodology

**DOI:** 10.1186/s40643-025-00934-0

**Published:** 2025-08-29

**Authors:** Rui Zhang, Han Wang, Li Wang, Zhiming Zheng

**Affiliations:** 1 College of Life Sciences, Anhui Medical University, Hefei, 230012 Anhui China; 2 Institute of Intelligent Machines, Hefei Institutes of Physical Science, Chinese Academy of Sciences, Hefei, 230031 China

**Keywords:** *Bacillus subtilis*, MK-7, Medium optimization, Response surface methodology

## Abstract

Menaquinone-7, a form of vitamin K_2_, plays a critical role in the treatment of hemorrhagic diseases caused by vitamin K deficiency and in the prevention of bone fractures. Microbial fermentation has emerged as a promising method for MK-7 production due to its high product optical purity and significant physiological activity. However, the current production efficiency of MK-7 remains insufficient to meet industrial demands. In this study, we employed a combination of single-factor experiments, Plackett-Burman design, steepest ascent experiments, and Box-Behnken design to optimize the fermentation medium for MK-7 production by *Bacillus subtilis* BS*-*Δ*ackA*. Through regression analysis and consideration of practical production constraints, the optimal fermentation medium parameters were determined as follows: 20 g/L sucrose, 20.7 g/L glycerol, 47.3 g/L soy peptone, 4 g/L yeast extract, and 1.9 g/L KH_2_PO_4_, 0.1 g/L MgSO_4_·7 H_2_O. Under these optimized conditions, the MK-7 yield reached 154.6 ± 1.32 mg/L. The experimental results demonstrated excellent stability and reliability, aligning closely with the model predictions. This study significantly enhances MK-7 production at the shake-flask fermentation level, providing valuable insights for large-scale industrial applications.

## Graphical Abstract

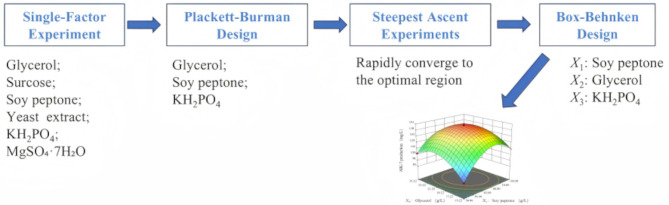

## Introduction

Menaquinone-7 (MK-7), a fat-soluble vitamin K derivative, is characterized by its redox activity. As a member of the vitamin K_2_ family, MK-7 shares the core structure of 2-methyl-1,4-naphthoquinone and is distinguished by a side chain comprising seven isoprenoid units(Vos et al. 2012; Li et al. 2018). MK-7 plays a crucial role in promoting blood coagulation(van Gorp et al. 2021) and has demonstrated significant efficacy in the prevention and treatment of osteoporosis(Shea and Holden 2012; Beulens et al. 2013). Recognized as a fourth-generation anti-osteoporosis drug(Shea and Holden 2012; Vermeer 2012), MK-7 also exhibits preventive and therapeutic effects against cardiovascular and cerebrovascular diseases associated with hypertension, hyperlipidemia, and atherosclerosis(Dalmeijer et al. 2012). Additionally, MK-7 has shown potential anti-tumor properties(Shi et al. 2018). However, humans cannot synthesize MK-7 endogenously and must obtain it from dietary sources such as meat, cheese, and fermented soybeans(Walther et al. 2013; Brudzynski and Maldonado-Alvarez 2018). The naturally low content of MK-7 in these foods is insufficient to meet the growing market demand. Consequently, the preparation and industrialization of MK-7 have emerged as a major research focus in recent years.

Currently, MK-7 is primarily produced through three methods: extraction from fermented foods, chemical synthesis, and microbial fermentation. However, the extraction of MK-7 from fermented foods is limited by its low concentration, which is insufficient to meet the therapeutic doses required for disease prevention and treatment. Chemical synthesis, although feasible, is associated with several drawbacks, including complex reaction steps, low yield(Isler et al. 1958; Shea and Holden 2012), the formation of low-activity cis-isomers(Min et al. 2003), the generation of substantial by-products(Isler et al. 1958), and potential environmental pollution. In contrast, microbial fermentation has emerged as the preferred method for MK-7 synthesis due to its mild reaction conditions and the production of highly bioactive compounds. Consequently, microbial fermentation is currently the primary focus of research in this field.

Bacteria serve as the primary producers of menaquinone, with different bacterial strains capable of synthesizing distinct types of menaquinone. Among these, the genus *Bacillus*, including *Bacillus subtilis* (*B. subtilis*) (Cui et al. 2020), *Bacillus natto* (*B. natto*) (Tsukamoto et al. 2001) and *Bacillus amyloliquefaciens* (*B. amyloliquefaciens*) (Wu and Ahn 2011), is regarded as a promising candidate for the industrial production of MK-7 (Beulens et al. 2009). Notably, *B.subtilis* is widely recognized as a safe food-grade microorganism and is commonly employed as a production strain for MK-7 due to its high resistance, lack of codon preference, well-characterized genetic background, extensive genetic modification tools, and advanced metabolic regulation techniques(Yang et al. 2016; Feng et al. 2017; Wang et al. 2019). Furthermore, MK-7 can constitute up to 96% of the total vitamin K_2_ synthesized by *B. subtilis*(Walther et al. 2013). However, the MK-7 yield of wild-type *B. subtilis* is insufficient for industrial applications. To address this limitation, researchers have employed strategies such as mutation breeding(Sato et al. 2001a, 2012), and genetic engineering(Xu and Zhang 2017; Liu et al. 2018; Yang et al. 2019) to enhance MK-7 production. While significant progress has been made, achieving the levels required for industrial-scale production remains a challenge(Lee et al. 2005). In previous studies, we disrupted the acetate biosynthesis pathway by knocking out the key gene *ackA* (acetate kinase) in *B.subtilis*. This genetic modification successfully redirected carbon flux from acetate synthesis toward MK-7 biosynthesis. The results demonstrated that, compared to the wild-type strain, the engineered strain BS-Δ*ackA* exhibited a significant 39.21% increase in MK-7 production, while acetate accumulation was completely eliminated (0 g/L vs. 5.56 g/L in the parent strain). Despite this improvement, further optimization of both strain performance and fermentation conditions is essential to achieve industrially viable productivity.

Optimization of the fermentation process is one of the most critical steps prior to large-scale industrial production(Wu and Ahn 2011). The efficient utilization of nutrients, particularly carbon, nitrogen, and phosphorus, in the fermentation medium is essential for enhancing the yield of target products in microbial fermentation(Cai and Zheng 2009). Previous studies have demonstrated that optimizing both the composition and concentration of the medium can significantly increase MK-7 production(Berenjian et al. 2012; Singh et al. 2015; Luo et al. 2017). For instance, Berenjian et al.(Berenjian et al. 2011) optimized the fermentation medium composition using response surface methodology, demonstrating that glycerol, soy peptone, yeast extract, and K_2_HPO_4_ significantly influenced MK-7 production. Similarly, Luo et al.(Luo et al. 2017) isolated *B. subtilis natto* from natto, and optimized the fermentation medium, fermentation conditions, and MK-7 extraction method. After incubating the strain for 72 h in a 5 L fermenter, they achieved an MK-7 yield of 32.2 mg/L. In addition, Hu et al.(Hu et al. 2017) optimized the concentration of carbon, nitrogen, and phosphorus sources in the fermentation medium of *B. subtilis* natto and investigated the effect of surfactants, leading to a substantial increase in MK-7 yield. These findings collectively highlight that optimizing the fermentation medium is an effective strategy for improving MK-7 production.

Response Surface Methodology (RSM) is a mathematical and statistical model used to establish a functional relationship between the target response and multiple variables(Khuri 2010). It is employed to simultaneously determine the optimal conditions for influencing factors among the variables under investigation, aiming to achieve an optimal response with a limited number of experiments(Jensen 2017). Due to its efficiency and precision, RSM has been widely adopted as an ideal tool for fermentation process development(Pawar and Rathod 2018; Suberu et al. 2019). For example, to enhance the spore yield of *B.subtilis* WHK-Z12, Chen et al.(Chen et al. 2010) optimized the culture medium via a combination of single-factor experiments, Plackett-Burman design, and central composite design. Under the optimal medium conditions, the spore yield reached 1.52 ± 0.06 × 10^10^ spores/mL at the shake flask level and 1.56 ± 0.07 × 10^10^ spores/mL in a 30-L fermenter following 40 h of cultivation. Similarly, Sun et al.(Sun et al. 2020) utilized RSM to maximize xylanase yield. The culture conditions were optimized through a Plackett-Burman design and RSM, and the enzyme source was identified using matrix-assisted laser desorption/ionization time-of-flight mass spectrometry (MALDI-TOF-MS). The results demonstrated a 2.1-fold and 1.4-fold increase in xylanase activity compared to the unoptimized medium and laboratory medium, respectively.

This study aimed to investigate the influence of different fermentation medium compositions on MK-7 production by *B.subtilis* and to determine the optimal medium composition for maximizing MK-7 yield. Using the pre-modified strain *B. subtilis* BS-Δ*ackA*, the fermentation process was optimized by evaluating MK-7 production as the key indicator. A combination of single-factor experiments, Plackett-Burman design, steepest ascent experiments, and Box-Behnken response surface methodology (RSM) was employed to identify the critical nutrients and their optimal concentrations for enhancing MK-7 production. The optimized process combination not only maximized the MK-7 production capacity of *B. subtilis* but also improved the conversion efficiency of fermentation raw materials, thereby reducing production costs. These findings provide a theoretical foundation for broadening the application of *B. subtilis* in the industrial-scale production of MK-7.

## Materials and methods

### Materials and instruments

The *B.subtilis* BS-Δ*ackA* was constructed and preserved by our laboratory.

Soy peptone was purchased from Biotopped (Beijing, China), while yeast extract and tryptone were obtained from OXOID (UK). Glycerol, glucose, NaCl, Na_2_HPO_4_, NaH_2_PO_4_, K_2_HPO_4_, KH_2_PO_4_, MgSO_4_·7H_2_O were purchased from Sinopharm (Shanghai, China). Organic reagents, including methanol, dichloromethane (HPLC grade), n-hexane, and isopropanol (analytical grade), were purchased from Rhawn Reagent (Shanghai, China). The MK-7 standard (purity ≥ 97%) was purchased from Aladdin (Catalog No. V302781, Shanghai, China). The standard was prepared by dissolving in methanol (1 mg/mL) and stored at -20℃ in amber vials to prevent degradation. Working solutions were freshly diluted before analysis.

The HPLC 1260 Infinity system was acquired from Agilent (USA).

### Experimental methods

#### Preparation of culture medium

The slant medium : yeast extract 5 g/L, tryptone 10 g/L, sodium chloride 10 g/L, agar 15–20 g/L ;

Seed medium : yeast extract 5 g/L, tryptone 10 g/L, sodium chloride10 g/L, pH 7.0 ;

Fermentation medium : 30 mL/L glycerol, 60 g/L soy peptone, 5 g/Lyeast extract,3 g/L K_2_HPO_4_, 0.5 g/L MgSO_4_·7H_2_O, pH 7.0.

All of the above cultures were based on sterilization at 115 °C for 15 min and cooling before use.

#### Activation and culture of Bacillus subtilis

A loop of *B. subtilis* BS-Δ*ackA* was aseptically transferred from the slant medium to a 250 mL shake flask containing 50 mL of LB liquid medium using a sterile inoculation loop. The culture was incubated at 37℃ with shaking at 220 rpm for 12 h. When the optical density at 600 nm (OD_600_) reached 5.5–6.0, the inoculum was transferred into a 500 mL shake flask containing 50 mL of LB fermentation medium at an inoculum rate of 8% (v/v). The fermentation medium consisted of 30 mL/L glycerol, 60 g/L soy peptone, 5 g/L yeast extract, 3 g/L K_2_HPO_4_, and 0.5 g/L MgSO_4_·7H_2_O, adjusted to pH 7.0. The culture was incubated at 40℃ with shaking at 250 rpm for 6 day. Samples were collected every day during the incubation period, and the cell density was determined by measuring the OD_600_ using a UV spectrophotometer.

#### Single-factor experiment for medium composition optimization

The single-factor experiment is a widely used optimization strategy in experimental research. It involves varying only one factor at a time while keeping all other factors constant, under the assumption that there is no interaction between the factors. This approach allows for the systematic investigation of the effect of different experimental levels on the results. In this study, to determine the optimal concentration range for each component of the fermentation medium, only one parameter in the initial medium composition was varied at a time, while all other factors were maintained constant.

To investigate the effects of delayed and available carbon sources on MK-7 production, glycerol was employed as the delayed carbon source at four concentrations: 25.22, 37.83, 50.44, and 63.05 g/L The impact of these concentrations on cell growth and MK-7 production was systematically evaluated. Additionally, glucose and sucrose were utilized as available carbon sources to examine their effects at different concentrations (10, 20, 30, and 40 g/L) on cell growth and MK-7 production.

To evaluate the effects of nitrogen sources on cell growth and MK-7 production, different concentrations of soy peptone (40, 50, 60, 70, and 80 g/L) and yeast extract (3, 4, 5, 6, 7, 8, 9, and 10 g/L) were employed.

The influence of phosphate sources, including K_2_HPO_4_, KH_2_PO_4_, Na_2_HPO_4_, and NaH_2_PO_4_ (3 g/L) on the growth of *B.subtilis* and the production of MK-7 were assessed. To further optimize the concentration of KH_2_PO_4_, a concentration gradient (0, 1, 2, 3, 4, 5, and 6 g/L) was tested for its effects on *B.subtilis* growth and MK-7 production. Additionally, the impact of metal ions (Mg²⁺, Cu²⁺, Fe²⁺, Mn²⁺, and Zn²⁺) at a concentration of 0.5 g/L on *B. subtilis* growth and MK-7 yield was investigated. To determine the optimal concentration of Mg^2+^, a concentration gradient (0, 0.1, 0.2, 0.3, 0.4, and 0.5 g/L) was examined for its effects on MK-7 production.

All experiments were conducted in triplicate, and the results were averaged for analysis.

#### Plackett-burman design of medium components

The Plackett-Burman design (PBD) is a two-level experimental methodology that efficiently identifies significant influencing factors among multiple variables with a minimal number of experiments (Oliveira et al. 2005).

Based on the optimization results from previous single-factor experiments, glycerol (A), sucrose (B), soy peptone (C), yeast extract (D), potassium dihydrogen phosphate (E), and magnesium sulfate heptahydrate (F) were selected as independent variables. Low (-1) and high (+ 1) levels were assigned to each factor to design the experimental matrix. A Plackett-Burman test with *N* = 12 was conducted to rapidly screen the medium components that exerted the greatest influence on MK-7 yield. The factors and their corresponding levels used in the PBD are presented in Table 1.

**Table 1:** Factors and levels design used in Plackett-Burman design (PBD)

Codes	Factors	Levels	
		-1	1
A	Glycerol(g/L)	25.22	50.44
B	Sucrose(g/L)	10	30
C	Soy peptone(g/L)	60	80
D	Yeast extract(g/L)	3	6
E	KH_2_PO_4_(g/L)	0.5	2
F	MgSO_4_·7H_2_O(g/L)	0	0.2

#### Steepest ascent experiment

After identifying the significant influencing factors through screening, the next step involves determining their optimal values. Prior to locating the region of maximum response, it is essential to construct an appropriate response surface fitting equation. Such an equation can accurately simulate the real system only in proximity to the optimal value. The steepest ascent method, a widely used strategy in response surface methodology (RSM) optimization experiments, facilitates the movement of significant factors identified in the Plackett-Burman design toward a near-optimal region under current operating conditions (Ekpenyong et al. 2021). Based on the Plackett-Burman design results, the three factors exerting the most significant influence on the target indicator are determined. The step size and direction of change are then designed according to their positive or negative effects. A positive effect is assigned when the estimated value increases, while a negative effect is assigned when it decreases. This approach enables rapid convergence to the optimal response region.

Based on the results of the Plackett-Burman design, the step size and direction of ascent for the significant influencing factors were determined, as outlined in Table 3. Non-significant factors were maintained at their initial concentrations. All experiments were conducted in triplicate, and the average values were recorded. The steepest ascent experiments were terminated when no further increase in response was observed.

Through the steepest ascent experiment, the point corresponding to the highest yield of MK-7 was identified as being in close proximity to the optimal region. This point was subsequently selected as the central point for the Box-Behnken design. The experimental levels for the steepest ascent test are presented in Table 2.

#### Box-behnken design

Based on the results of the Plackett-Burman design and steepest ascent experiments, a three-factor, three-level response surface methodology (RSM) was employed using the Box-Behnken design (BBD) in Design-Expert 8.0.6 software to optimize the fermentation process parameters for MK-7 production. A total of 17 experimental runs were designed, with MK-7 production as the response variable. The independent variables, namely soy peptone (A), glycerol (B), and KH_2_PO_4_ (C), were evaluated at three coded levels: −1, 0, and + 1. Each experiment was performed in triplicate. The factors and their corresponding levels in the BBD are presented in Table 3.

**Table 2:** Factors and levels of Box-Behnkent design

Codes	Factors	Levels		
		-1	0	1
A	Soy peptone(g/L)	30	45	60
B	Glycerol(g/L)	15.22	20.22	25.22
C	KH_2_PO_4_(g/L)	1.8	1.9	2

**Table 3:** Experimental level settings for the steepest ascent method

Codes	Soy peptone g/L	Glycerol g/L	KH_2_PO_4_ g/L
1	75	30.22	1.7
2	60	25.22	1.8
3	45	20.22	1.9
4	30	15.22	2.0
5	15	10.22	2.1

#### Extraction and quantification of MK-7

Extraction of MK-7: At the end of fermentation, 1 mL of fermentation broth was collected and mixed with 3 mL of a solvent mixture consisting of n-hexane and isopropanol (2:1, v/v). The mixture was subjected to ultrasonication for 30 min, followed by vigorous vortexing at 2,500 rpm for an additional 30 min. The organic phase (upper layer) was then filtered through a 0.22 μm organic membrane to obtain the MK-7 extract. All extraction procedures were performed under light-protected conditions to prevent photodegradation.

Quantification of MK-7 by High-Performance Liquid Chromatography (HPLC): MK-7 production was quantified using an HPLC system equipped with a UV detector (SPD-16) and a reversed-phase C-18 column (VP-ODS, 4.6 mm×250 mm). The mobile phase consisted of methanol and dichloromethane (4:1, v/v) at a flow rate of 1 mL/min. The column temperature was maintained at 35 °C, and the detection wavelength was set at 248 nm. The injection volume was 20 µL. A calibration curve was established within the range of 0–150 mg/L, demonstrating a strong linear relationship between MK-7 concentration and peak area (*R*^2^ = 0.999).

#### Determination of cell density and pH

During fermentation, samples were collected at designated time intervals to assess cell density. The samples were appropriately diluted with deionized water, and cell density was measured using an ultraviolet-visible spectrophotometer (UV-1200, Shanghai, China) at an optical density of 600 nm (OD_600_). Concurrently, pH values were directly measured using a calibrated laboratory pH meter (Hanna Instruments, USA).

### Data processing and analysis

Regression analysis and analysis of variance (ANOVA) were performed using Design-Expert 8.06 software. The optimal conditions for each factor were determined and validated based on the prediction results. Statistical significance was defined as (*P* < 0.05). Data are presented as the mean ± standard error of the mean (SEM) from triplicate experiments ((*n* = 3)). Graphical representations were generated using Origin 2024 software.

## Results and discussion

### Single-factor experiment

#### Effect of carbon source

Optimization of medium composition is a critical strategy for enhancing the yield of MK-7. As a key component of the medium, carbon serves as the primary energy source for microorganisms, significantly influencing cell growth and the production of both primary and secondary metabolites.

**Fig. 1:**
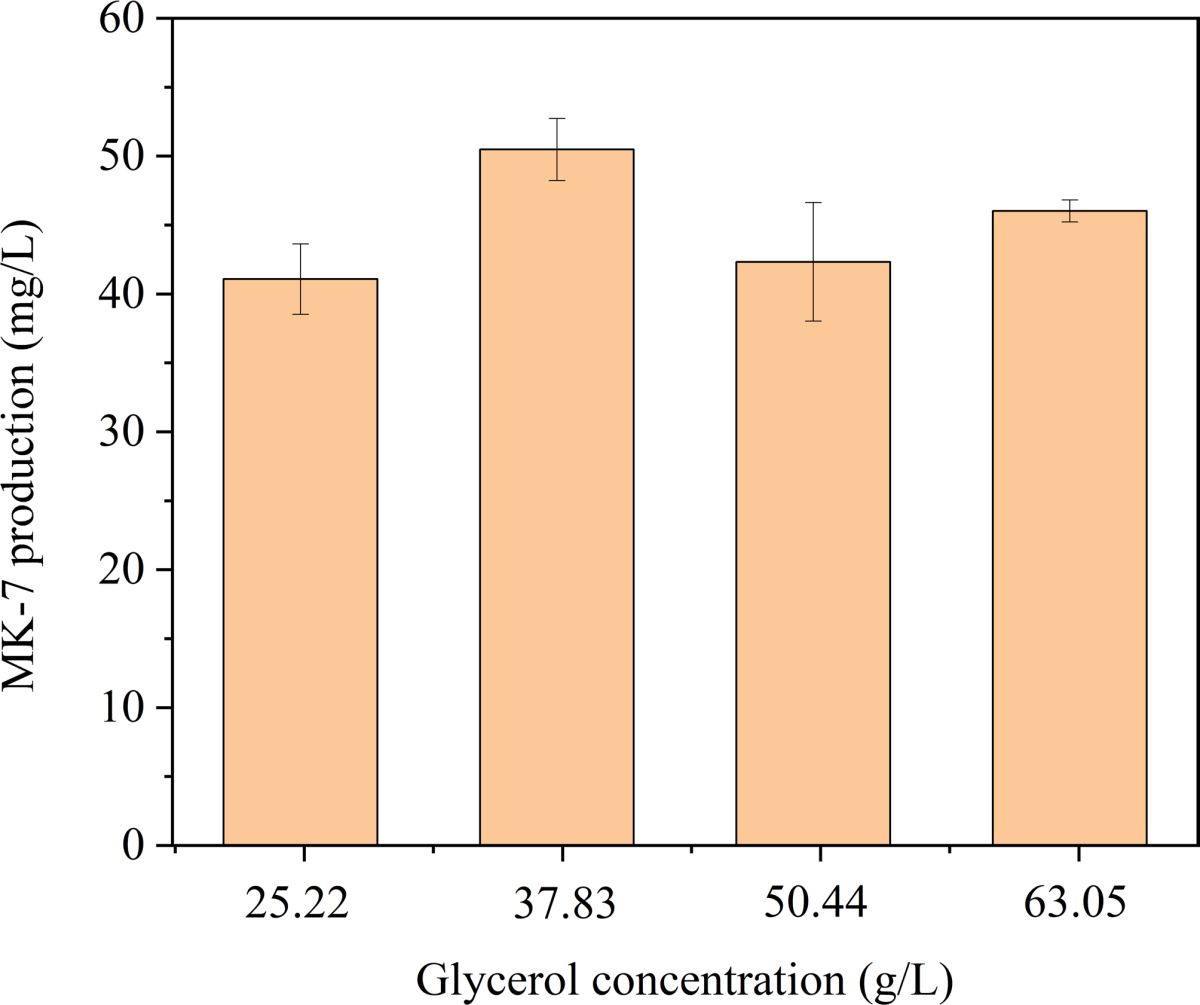
Effect of glycerol concentrations on BS-Δ*ackA* fermentation

Previous studies have identified glycerol as the optimal carbon source for MK-7 synthesis(Sato et al. 2001a, b). In this study, the effect of varying glycerol concentrations on MK-7 production by *B.subtilis* was investigated. As shown in Fig. 1, MK-7 production initially increased with glycerol concentration but subsequently declined, suggesting that excessive glycerol is not efficiently utilized by the cells and may slightly inhibit MK-7 synthesis. This observation aligns with the findings of Wu et al.(Zhao et al. 2021). The inhibitory effect may be attributed to the hypertonic nature of glycerol, which can alter cell membrane permeability and impair the transmembrane transport of metabolites. The maximum MK-7 yield of 50.47 ± 2.25 mg/L was achieved at a glycerol concentration of 37.83 mg/L, indicating that this concentration is optimal for MK-7 production.

**Fig. 2:**
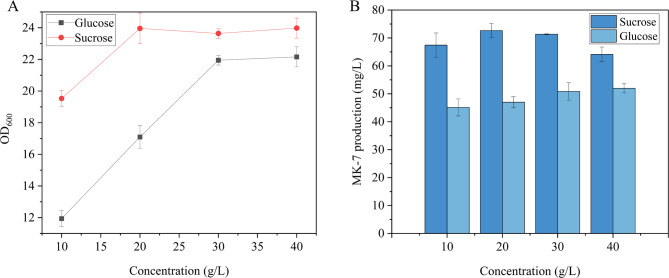
Effect of sucrose and glucose on BS-Δ*ackA* fermentation. (**A**) Influence of glucose and sucrose concentrations on OD_600_. (**B**) Effect of glucose and sucrose concentrations on MK-7 yield

When glycerol was employed as the primary carbon source, the effects of different concentrations of glucose and sucrose (10, 20, 30, and 40 g/L) on cell growth and MK-7 production were investigated. As illustrated in Fig. 2A and B, sucrose as a carbon source resulted in higher cell density and MK-7 production compared to glucose at all concentrations tested. With increasing sucrose concentration, cell density initially increased and then decreased, yet remained higher than that of the glucose group. The maximum cell density of 23.96 ± 0.97 was observed at a sucrose concentration of 20 g/L, accompanied by an MK-7 production of 72.65 ± 2.61 mg/L, representing a 35.3% increase compared to the control (20 g/L glucose). In contrast, while cell density increased significantly with increasing glucose concentration, MK-7 production showed only marginal improvement.

The observed differences can be attributed to the following factors:

Substrate Utilization: Sucrose, a disaccharide, is metabolized by hydrolysis into glucose, thereby mitigating the inhibitory effects associated with high glucose concentrations.

Metabolic Pathway Inhibition: Glucose inhibits the *glpF-glpK* operon, which is involved in the catabolism of glycerol and glycerol-3-phosphate in the glycerol isomerization pathway of *B. subtilis* (Holmberg and Rutberg 1991).

By-product Formation: When glucose is used as a substrate, fermentation by-products such as acetic acid, ethanol, lactic acid, ethylidene glycol, and butanediol are generated (Oliveira et al. 2005; Białkowska et al. 2016). These by-products are derived from pyruvate, a key intermediate in the glycolytic pathway. Since pyruvate also serves as a precursor for MK-7 side-chain synthesis, the diversion of pyruvate to by-product formation competes with the MK-7 biosynthesis pathway, resulting in reduced MK-7 yields (Yang et al. 2019).

These factors collectively explain the lower MK-7 production observed when glucose was used as the carbon source.

In conclusion, the combination of 37.83 mg/L glycerol and 20 g/L sucrose was found to enhance both cell growth and MK-7 synthesis, and was therefore selected as the optimal carbon source composition for the fermentation medium.

#### Effect of nitrogen source

Nitrogen source is a critical nutrient for microbial growth, reproduction, and metabolic activities, serving as a fundamental component for the synthesis of cellular substances (e.g., amino acids, proteins, and nucleic acids) and nitrogen-containing metabolites. It plays an essential role in supporting microbial life processes. Previous studies have demonstrated that soy peptone is the optimal nitrogen source for enhancing both cell growth and MK-7 production(Li et al. 2023).

**Fig. 3:**
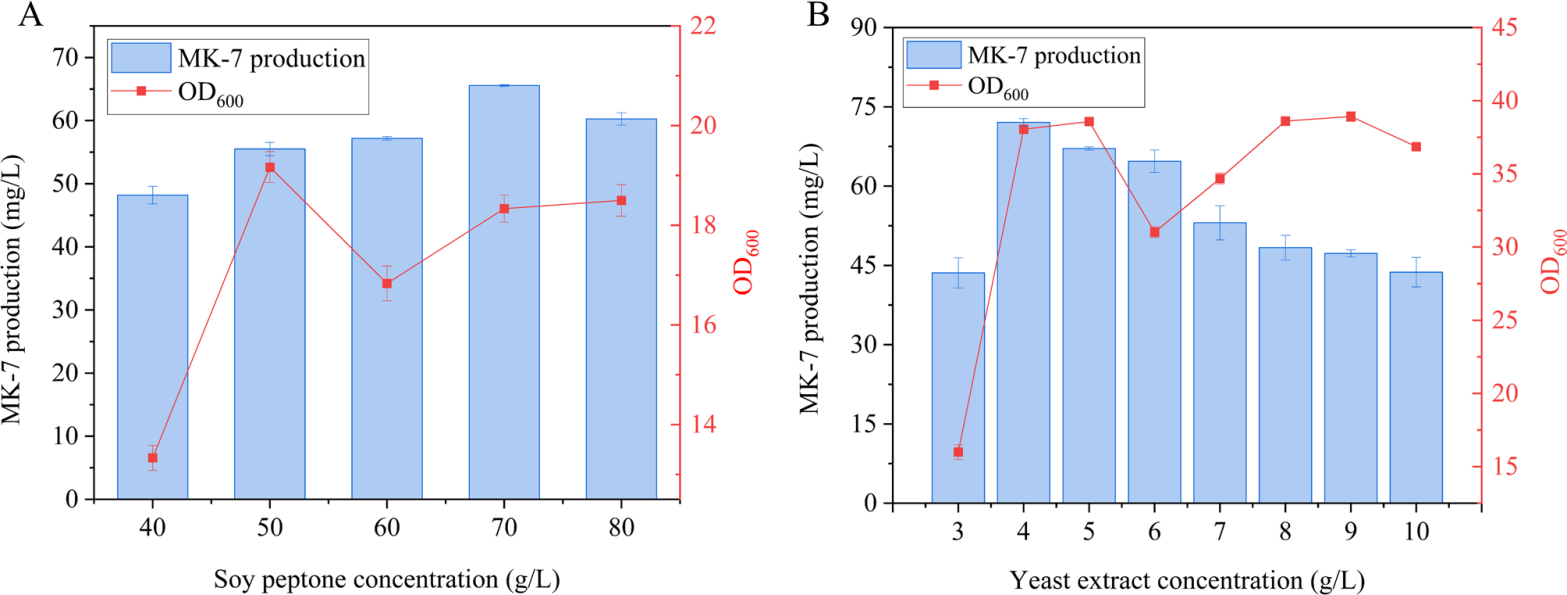
Effect of nitrogen source on BS-Δ*ackA* fermentation. (**A**) Growth curve of BS-Δ*ackA* and MK-7 production at varying concentrations of soy peptone. (**B**) Growth curve of BS-Δ*ackA* and MK-7 production at varying concentrations of yeast extract

To determine the optimal concentration of soy peptone in the original medium, a series of experiments were conducted using different concentration gradients. As illustrated in Fig. 3A, the highest cell density (18.33 ± 0.27) and MK-7 production (65.55 ± 0.14 mg/L) were achieved when 70 g/L of soy peptone was utilized as the nitrogen source. These findings indicate that 70 g/L of soy peptone is the optimal concentration for both cell growth and MK-7 production in strain BS-Δ*ackA*. However, exceeding this concentration led to a notable inhibition of MK-7 synthesis, a phenomenon consistent with previous studies on nitrogen-induced inhibition in biosynthesis processes(Adinarayana and Ellaiah 2002). This inhibitory effect may be attributed to the complex composition of soy peptone, which contains various amino acids and organic compounds. These components may compete with other essential nutrients, such as carbon sources and metal ions, thereby disrupting metabolic balance and reducing MK-7 production.

Complex nitrogen sources are widely employed in *B.subtilis* fermentation processes, with studies demonstrating that a combination of yeast extract and soy peptone significantly enhances MK-7 yields(Vos et al. 2012). Yeast extract, in particular, has been reported as a readily utilizable nitrogen source for bacterial growth(Sato et al. 2012), making it a valuable nutritional supplement for the production of value-added metabolites. To further improve MK-7 production in strain BS-Δ*ackA*, this study investigated the effect of yeast extract concentration (ranging from 3 to 10 g/L) on both cell density and MK-7 yield.

As depicted in Fig. 3B, the maximum cell density (38.92 ± 0.33) was achieved at a yeast extract concentration of 9 g/L. In contrast, MK-7 production peaked at 72.07 ± 0.71 mg/L when the yeast extract concentration was 4 g/L. Notably, MK-7 yield gradually declined as the yeast extract concentration increased beyond this optimal level. These results suggest that only an appropriate concentration of yeast extract is conducive to enhancing MK-7 production. The inhibitory effect observed at higher yeast extract concentrations may be attributed to the increased metabolic burden and elevated osmotic pressure, both of which adversely affect bacterial physiology and metabolic efficiency, ultimately reducing MK-7 synthesis.

Based on these findings, 70 g/L of soy peptone and 4 g/L of yeast extract were selected as the optimal nitrogen sources for subsequent experiments.

#### Effect of inorganic salts

Inorganic salts, particularly certain metal ions, play a critical role in microbial growth and metabolism. These compounds are integral to various metabolic processes and serve as coenzyme centers in numerous enzyme systems.

**Fig. 4:**
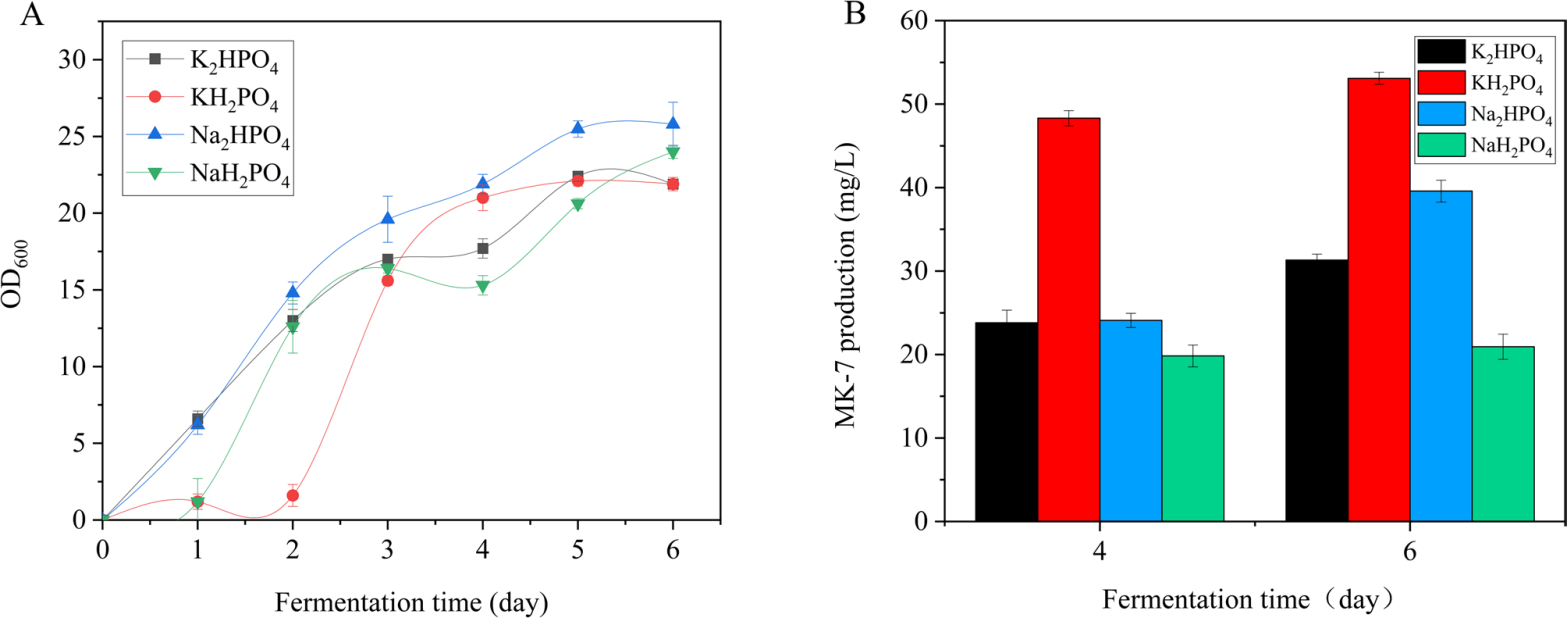
Effect of inorganic salts on the fermentation of BS-ΔackA. (**A**) Growth curves over a 6-day fermentation period with varying concentrations of phosphate added to the fermentation medium. (**B**) MK-7 yields after 4 and 6 days of fermentation with different phosphate concentrations in the fermentation medium

To investigate the influence of inorganic salts on MK-7 production, K_2_HPO_4_, KH_2_PO_4_, Na_2_HPO_4_, and NaH_2_PO_4_ were individually added to the fermentation medium while keeping other components constant. As shown in Fig. 4A, regardless of the phosphate source employed, BS-Δ*ackA* exhibited a typical growth pattern consisting of an initial lag phase, followed by rapid exponential growth, and eventual stabilization. Notably, cultures supplemented with KH_2_PO_4_ displayed a more pronounced exponential phase, with a sharp increase in OD_6_₀₀ between days 2 and 4. This suggests that BS-Δ*ackA* efficiently metabolizes KH_2_PO_4_ for growth, with potassium ions potentially playing an auxiliary role in enhancing cellular metabolism. In contrast, Na_2_HPO_4_ demonstrated a stronger growth-promoting effect, sustaining higher OD_6_₀₀ values during the late fermentation stage (after day 3). This indicates that the phosphorus and sodium ion composition of Na_2_HPO_4_ better supports bacterial proliferation and physiological activity, likely due to its alignment with the metabolic demands of BS-Δ*ackA*. The growth-supporting effects of K_2_HPO_4_ and NaH_2_PO_4_ were comparatively moderate, with both growth rates and final biomass yields falling between those observed with Na_2_HPO_4_ and KH_2_PO_4_. This suggests that the specific cation (K⁺/Na⁺) and phosphate anion combinations in these salts exert a more balanced, albeit slightly less stimulatory, influence on bacterial development relative to the other phosphate sources tested.

In terms of MK-7 biosynthesis, however, the addition of KH_2_PO_4_ resulted in significantly higher MK-7 production (53.1 ± 0.72 mg/L) compared to other phosphates (Fig. 4B). This phenomenon may be attributed to the role of phosphates in modulating the biosynthesis pathway of MK-7, potentially by regulating the activity of key enzymes or the concentration of metabolites. Based on these findings, KH_2_PO_4_ was selected for further optimization experiments.

**Fig. 5:**
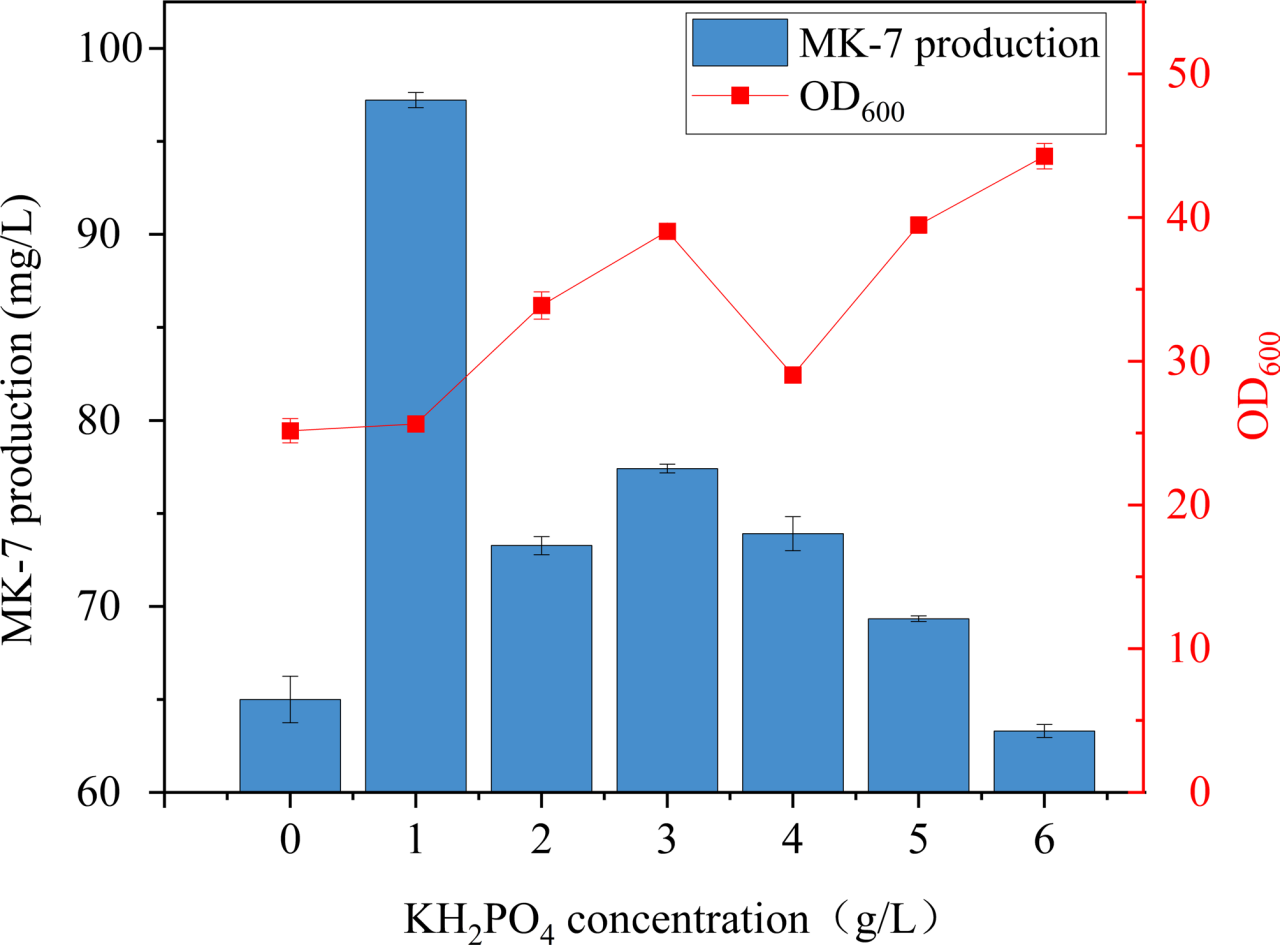
Growth levels of the strains and MK-7 production at different concentrations of KH_2_PO_4_

To investigate the effect of KH_2_PO_4_ on biomass and MK-7 production in BS-Δ*ackA*, different concentration gradients (0, 1, 2, 3, 4, 5, and 6 g/L) were tested. The results revealed that MK-7 production reached a maximum of 97.23 ± 0.41 mg/L at a KH_2_PO_4_ concentration of 1 g/L, which was significantly higher than in the other tested KH_2_PO_4_ concentrations (0, 2, 3, 4, 5, and 6 g/L) (Fig. 5). However, when the KH_2_PO_4_ concentration exceeded a specific threshold, a noticeable decline in MK-7 production was observed. Based on these results, a KH_2_PO_4_ concentration of 1 g/L was selected for subsequent experiments.

Metal ions play a pivotal role in microbial culture systems, significantly influencing microbial growth, metabolite synthesis, enzyme activity(Prejano et al. 2020), and cellular signal transduction(Landriscina et al. 2001). Consequently, precise regulation of the concentration and types of metal ions is essential to ensure optimal microbial growth and metabolic activity.

**Fig. 6:**
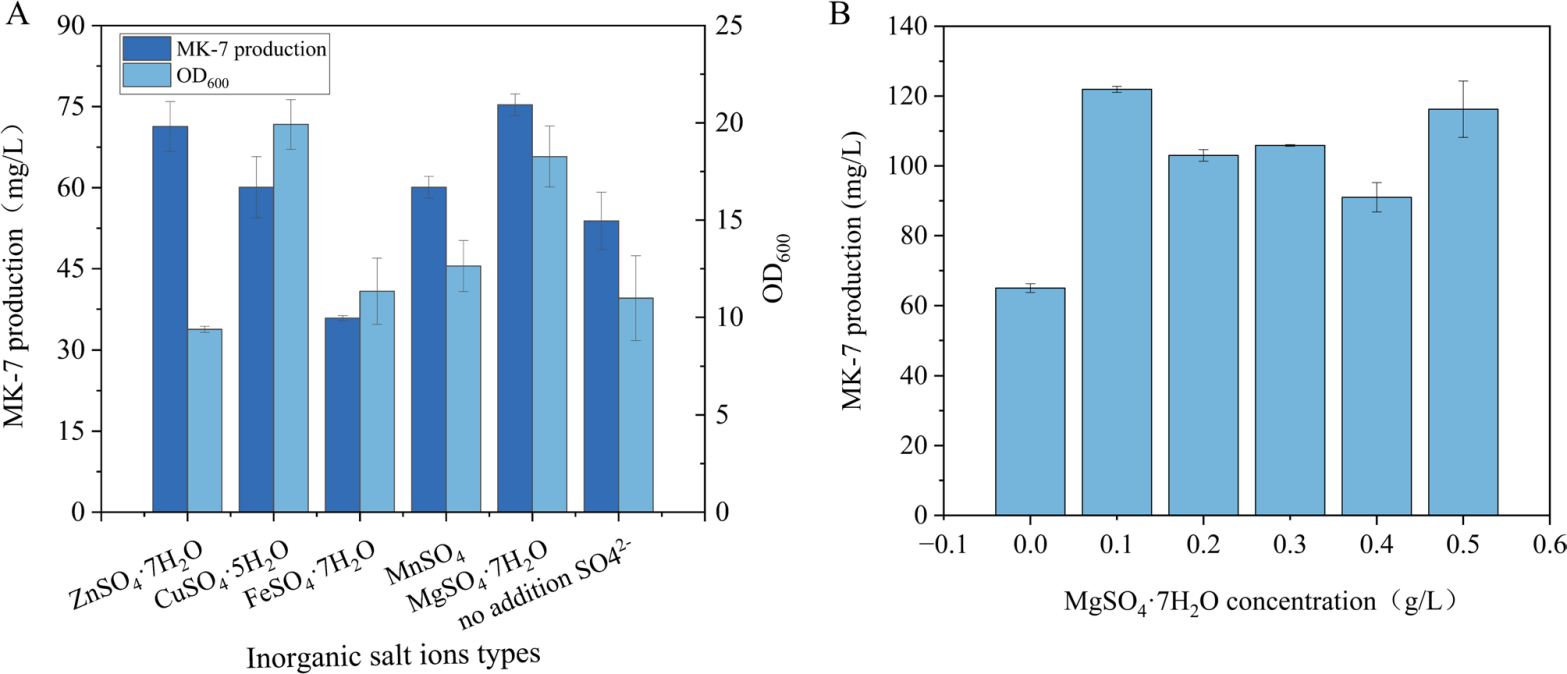
Influence of metal ions on the fermentation performance of *B.subtilis* BS-ΔackA. (**A**) Impact of various metal ions on MK-7 production by *B.subtilis* BS-Δ*ackA*. (**B**) Effect of different MgSO_4_·7 H_2_O concentrations on MK-7 production by *B.subtilis* BS-Δ*ackA*

This study investigated the effects of metal ions, including Mg²⁺, Cu²⁺, Fe²⁺, Mn²⁺, and Zn²⁺, on the growth of *B.subtilis* BS-Δ*ackA* and the production yield of MK-7. As illustrated in Fig. 6A, the supplementation of Mg²⁺ in the fermentation medium significantly enhanced MK-7 production compared to other metal ions. This effect is likely attributed to the role of magnesium ions as essential cofactors in numerous enzymatic reactions, including energy metabolism, protein synthesis, and cellular signaling(Lioy et al. 2012; Gröber et al. 2015). Furthermore, Mg²⁺ facilitates ATP synthesis at optimal concentrations(Wu et al. 2014). In *B.subtilis*, magnesium ions may modulate growth and metabolic processes by influencing intracellular signaling pathways and gene expression(De Eustaquio-Campillo et al. 2017). Additionally, the regulatory mechanisms of magnesium ions in microbial growth and metabolism may involve their impact on phosphate homeostasis(Pontes and Groisman 2018). Mg²⁺ also plays a critical role in maintaining intra- and extracellular magnesium ion homeostasis through its interaction with membrane transporter proteins, such as TRPM6 and TRPM7, thereby affecting bacterial fitness and virulence(Zhou and Clapham 2009; Robak et al. 2016).

To optimize MK-7 production, the effects of varying concentrations of MgSO_4_·7 H_2_O (0, 0.1, 0.2, 0.3, 0.4, and 0.5 g/L) were evaluated. The results demonstrated that the highest MK-7 yield (121.95 ± 0.88 mg/L) was achieved at a MgSO_4_·7 H_2_O concentration of 0.1 g/L (Fig. 6B). Consequently, this concentration was selected for subsequent experiments.

### Screening of significant factors affecting MK-7 fermentation using plackett-burman design

While single-factor experiments are useful for examining the influence of individual factors on the response variable, they are unable to account for interactions among multiple factors. The Plackett-Burman design, on the other hand, is an efficient statistical tool for identifying the primary factors influencing a process from a large set of variables.

In this study, a Plackett-Burman design was employed to screen the significant factors affecting MK-7 production by *B.subtilis*. The factors investigated included glycerol (A), sucrose (B), soybean peptone (C), yeast extract (D), KH_2_PO_4_ (E), and MgSO_4_·7 H_2_O (F), with MK-7 production as the response variable. Table 4 presents the effects of 12 different combinations of these six factors at two levels on the yield of MK-7. Table 5 summarizes the analysis of variance (ANOVA) results for the experimental data.

**Table 4:** Plackett-Burman design scheme and results

Group	A	B	C	D	E	F	MK-7 production mg/L
	Glycerol	Sucrose	Soybean peptone	Yeast extract	KH_2_PO_4_	MgSO_4_·7 H_2_O	
1	-1	1	1	1	-1	-1	80.369 ± 0.58
2	1	1	-1	1	1	1	108.229 ± 1.05
3	-1	1	-1	1	1	-1	130.511 ± 1.02
4	1	-1	-1	-1	1	-1	112.903 ± 1.90
5	1	1	-1	-1	-1	1	109.425 ± 1.79
6	-1	-1	-1	-1	-1	-1	105.707 ± 0.17
7	1	-1	1	1	-1	1	53.166 ± 1.56
8	-1	-1	1	-1	1	1	88.219 ± 1.28
9	1	-1	1	1	1	-1	58.346 ± 1.51
10	-1	-1	-1	1	1	1	136.864 ± 1.45
11	1	1	1	-1	-1	-1	65.405 ± 1.20
12	-1	1	1	-1	1	1	85.988 ± 1.01

Note: +1 represents a high level of the factor and − 1 represents a low level of the factor, the exact values of which are given in 2.2.4

The experimental data presented in Table 4 were subjected to linear regression analysis using Design-Expert 8.0 software. The optimal regression equation for MK-7 production by *B. subtilis* was determined as follows:

1 $$\text{Y}=94.59-10.02\text{A}+2.06\text{B}-22.68\text{C}-0.013\text{D}+2.77\text{E}+2.39\text{F}$$

The model yielded an *F*-value of 11.42, with a corresponding *P*-value of 0.0086 (*P* < 0.01), indicating that the regression model is highly statistically significant. Furthermore, the coefficient of determination (*R*²=0.9320) demonstrated that 93.20% of the variability in the experimental data can be explained by Eq. 1. This high *R*² value confirms that the regression model is well-fitted and representative of the experimental results. Based on these findings, the regression model is considered stable and reliable for evaluating the influence of each experimental factor on the response value (MK-7 production).

The analysis of variance (ANOVA) results presented in Table 5 revealed the relative influence of the experimental factors on the response value (Y). The order of their impact was determined as follows: soy peptone (C) > glycerol (A) > KH_2_PO_4_ (E) > MgSO_4_·7 H_2_O (F) > sucrose (B) > yeast extract (D). Based on these findings, the three most significant factors—soy peptone, glycerol, and KH_2_PO_4_—were selected for further optimization using the steepest ascent experiment.

**Table 5:** The result of the analysis of Plackett-Burman design

Source	Sum of squares	df	Mean square	F-value	*p*-value	effect sequence
Model	7587.19	6	1264.53	11.42	0.0086	
A-Glycerol	1203.71	1	1203.71	10.87	0.0215	2
B-Sucrose	50.93	1	50.93	0.46	0.5277	5
C-Soy peptone	6171.96	1	6171.96	55.76	0.0007	1
D-Yeast extract	2.184E-003	1	2.184E-003	1.973E-005	0.9966	6
E-KH_2_PO_4_	92.18	1	92.18	0.83	0.4033	3
F-MgSO_4_·7 H_2_O	68.40	1	68.40	0.62	0.4674	4
Residual	553.47	5	110.69			
Cor total	8140.66	11				
*R*^2^ = 0.9320;*R*^2^_adj_ = 0.8504						

### Steepest ascent experiment

Based on the results obtained from the Plackett-Burman design experiments, it was observed that the three factors significantly influencing the production of MK-7 by *B.subtilis* exhibited distinct correlations with the response value. Specifically, soy peptone and glycerol demonstrated a negative correlation with the response value, whereas KH_2_PO_4_ exhibited a positive correlation. Consequently, in the design of the steepest ascent experiment, the concentrations of soy peptone and glycerol were progressively decreased, while the concentration of KH_2_PO_4_ was incrementally increased.

The experimental results (Table 6) demonstrated that the yield of MK-7 initially increased and subsequently decreased in response to variations in the concentrations of the three key factors: soy peptone, glycerol, and KH_2_PO_4_. The highest values for all three response indices were achieved under the conditions of the third experimental group. Consequently, the factor concentrations corresponding to the third experimental group were selected as the central point for the response surface methodology (RSM) design.

**Table 6:** Design and results of the steepest ascent experiment

Codes	Soy peptone g/L	Glycerol g/L	KH_2_PO_4_ g/L	MK-7 production mg/L
1	75	30.22	1.7	80.40 ± 1.30
2	60	25.22	1.8	60.20 ± 1.30
3	45	20.22	1.9	99.88 ± 2.71
4	30	15.22	2.0	79.64 ± 1.90
5	15	10.22	2.1	9.48 ± 0.34

### Analysis of box-behnken results

Based on the results of the steepest ascent experiment, a response surface analysis was conducted using the Box-Behnken experimental design. This design evaluated the three significant factors—soybean peptone (*X*_1_ ), glycerol (*X*_2_), and KH_2_PO_4_ (*X*_3_)—at three levels, with the yield of MK-7 as the response variable. The experimental results are presented in Table 7.

#### Regression equation and variance analysis

A quadratic multiple regression model was fitted to the experimental data presented in Table 7 using Design Expert 8.0.6 software. The resulting regression equation, which describes the relationship between the yield of MK-7 and the three independent variables (soybean peptone, *X*_1_ ; glycerol, *X*_2_; and KH_2_PO_4_, *X*_3_), is as follows:

2 $$\begin{array}{l}\text{Y}=146.27+7.90{X}_{1}+4.00{X}_{2}-7.24{X}_{3}-\\6.30{X}_{1}{X}_{2}-5.54{X}_{1}{X}_{3}-2.33{X}_{2}{X}_{3}-26.79{{X}_{1}}^{2}-20.17{{X}_{2}}^{2}-\\20.19{{X}_{3}}^{2}\end{array}$$

The analysis of variance (ANOVA) for the regression model is summarized in Table 8.

As shown in Table 8, the F-value of the composite score model is 63.28 with a *p*-value of < 0.0001, demonstrating that the model is highly significant and statistically valid. Additionally, the lack of fit test yielded a *p*-value of 0.5813 (*p* > 0.05), indicating that it is not significant, which suggests that unknown factors have minimal influence on the experimental results.

The model exhibited a coefficient of determination (*R*^2^) of 0.9879 and an adjusted *R*^2^ (*R*^2^_adj_) of 0.9722, indicating that the model explains 97.22% of the total variation in the composite score of chytrid fermentation, while only approximately 2.78% of the variation remains unpredicted. Furthermore, the coefficient of variation (*CV*) of the model is 3.38%, reflecting a relatively good fit, low experimental error, and strong agreement between the predicted and actual experimental values. These results demonstrate that the model equation is reliable for analyzing and predicting the effects of various factors on the production of MK-7 by *B. subtilis*.

According to Table 8, the primary factors soy peptone (*X*_1_) exhibited highly significant effects on MK-7 yield (*P <* 0.001), indicating that even minor variations in their concentrations could substantially influence the rate of product formation. The primary factor glycerol (*X*_2_) and KH_2_PO_4_ (*X*_3_) also demonstrated a significant effect on MK-7 yield (*P* < 0.05). Furthermore, the quadratic terms (*X*_1_^2^, *X*_2_^2^, *X*_3_^2^) were found to have highly significant effects on MK-7 production during BS-Δ*ackA* fermentation (*P <* 0.001). These results suggest that the relationship between the factors and MK-7 production is better described by a quadratic model rather than a simple linear relationship.

The interaction terms *X*_1_* × *_2_ and *X*_1_* × *_3_ exhibited highly significant effects on MK-7 production during *B.subtilis* fermentation (*P* < 0.05), indicating strong interactions between soy peptone concentration and glycerol concentration, as well as between soy peptone concentration and KH_2_PO_4_ concentration. In contrast, the interaction term *X*_*2*_* × *_3_ showed no significant effect, suggesting that the interaction between glycerol concentration and KH_2_PO_4_ concentration did not significantly influence MK-7 production. Based on the F-value analysis, the order of influence of each factor on the composite score was as follows: *X*_1_ > *X*_3_ > *X*_2_ (soy peptone > KH_2_PO_4_ > glycerol).

**Table 7:** Design and results of Box-Behnken experiments

Run	X_1_ Soy peptone	X_2_ Glycerol	X_3_ KH_2_PO_4_	MK-7 production
				mg/L
1	0	-1	-1	109.02 ± 0.66
2	0	1	-1	121.60 ± 1.32
3	0	0	0	145.70 ± 2.38
4	-1	0	1	91.76 ± 0.4
5	0	0	0	139.39 ± 2.90
6	1	0	-1	117.89 ± 0.00
7	-1	-1	0	81.15 ± 0.63
8	-1	1	0	101.85 ± 1.17
9	-1	0	-1	90.85 ± 0.19
10	1	-1	0	109.37 ± 1.17
11	1	1	0	104.86 ± 2.00
12	0	-1	1	94.87 ± 0.62
13	0	1	1	98.13 ± 0.98
14	0	0	0	148.06 ± 1.56
15	1	0	1	96.65 ± 2.65
16	0	0	0	149.70 ± 0.21
17	0	0	0	148.48 ± 0.34

**Table 8:** Box-Behnken test regression model and variance analysis

Source	Sum of squares	df	Mean square	F-value	*p*-value	Significant
Model	8539.21	9	948.81	63.28	< 0.0001	**
*X*_1_-Soy peptone	498.65	1	498.65	33.26	0.0007	**
*X*_2_-Glycerol	128.24	1	128.24	8.55	0.0222	*
*X*_3_-KH_2_PO_4_	419.78	1	419.78	28.00	0.0011	*
*X*_1_ *X*_2_	158.89	1	158.89	10.60	0.0140	*
*X*_1_ *X*_3_	122.66	1	122.66	8.18	0.0243	*
*X*_2_ *X*_3_	21.72	1	21.72	1.45	0.2679	
*X*_1_^2^	3021.46	1	3021.46	201.51	< 0.0001	**
*X*_2_^2^	1713.05	1	1713.05	114.25	< 0.0001	**
*X*_3_^2^	1716.45	1	1716.45	114.48	< 0.0001	**
Residual	104.96	7	14.99			
Lack of fit	37.44	3	12.48	0.74	0.5813	no significant
Pure error	67.51	4	16.88			
Cor total	8644.23	16				
*R* ^2^	0.9879					
Adjusted *R*^2^	0.9722					
Predicted *R*^2^	0.9185					

Note: ‘*’ indicates that the effect of the factor on the response value is significant (*p* < 0.05) and ‘**’ indicates that the effect of the factor on the response value is highly significant (*p* < 0.001)

#### Interaction analysis between two factors

**Fig. 7:**
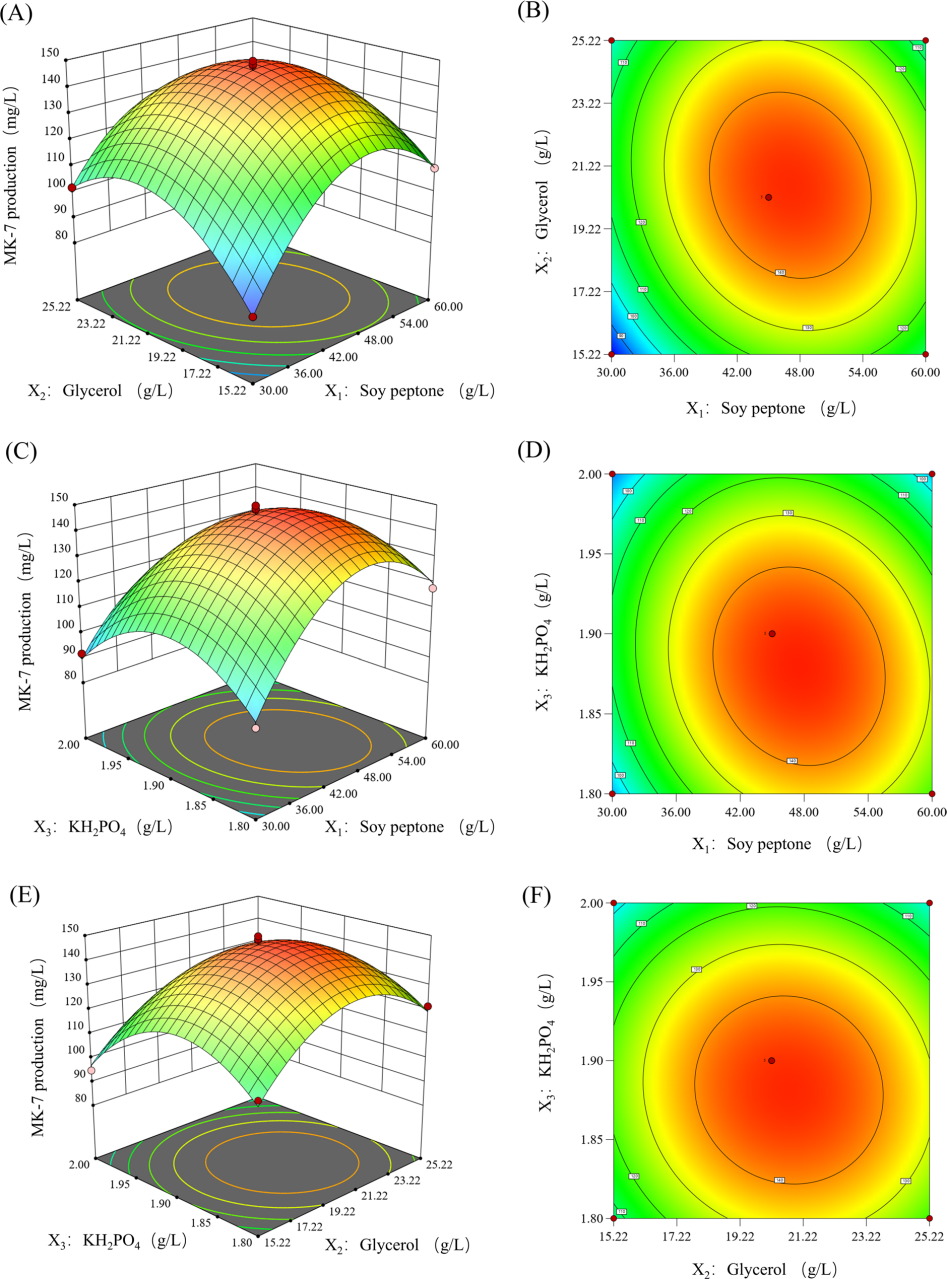
Response surface and contour plots illustrating the effects of nutrient interactions on MK-7 production by BS-ΔackA. (**A**) Response surface plot for the interaction between soy peptone concentration and glycerol concentration. (**B**) Contour plot for the interaction between soy peptone concentration and glycerol concentration. (**C**) Response surface plot for the interaction between soy peptone concentration and KH_2_PO_4_ concentration. (**D**) Contour plot for the interaction between soy peptone concentration and KH_2_PO_4_ concentration. (**E**) Response surface plot for the interaction between glycerol concentration and KH_2_PO_4_ concentration. (**F**) Contour plot for the interaction between glycerol concentration and KH_2_PO_4_ concentration

To visualize the interactions among the three factors more intuitively, the maximum response value was identified. Based on the regression equation and the ANOVA table of the regression model, response surface plots and their corresponding contour plots were generated using Design-Expert 8.0.6 software. These plots were constructed by varying two independent variables within the experimental range while holding the third variable at its zero level (Fig. 7). Each response surface plot illustrates the effect of the interaction between two independent variables on MK-7 production during BS-Δ*ackA* fermentation, under the condition that one variable remains at its optimal level. The significance of the interaction is indicated by the steepness of the response surface and the uneven distribution of contour lines. Furthermore, the closer the contour lines resemble an ellipse, the stronger the interaction between the two factors.

As shown in Fig. 7 (A)-(D), when one variable is held constant, the influence of the other two factors on the response value exhibits a parabolic trend, with a distinct maximum point observed. The response value initially increases and then decreases, resulting in a convex response surface. The contour lines in these plots are elliptical, indicating a significant interaction between factors *X*_1_* × *_2_ and *X*_1_* × *_3_. These findings are consistent with the ANOVA results presented in Table 8.

In contrast, the contour plots (E)-(F) in Fig. 7 are nearly circular, despite the relatively steep response surface. This suggests that the interaction between glycerol and KH_2_PO_4_ is not significant, which is also in agreement with the ANOVA results in Table 8.

#### Model validation experiments

The optimal fermentation medium parameters predicted by the statistical analysis were as follows: sucrose 20 g/L, glycerol 20.65 g/L, soybean peptone 47.38 g/L, yeast extract 4 g/L, and KH_2_PO_4_ 1.88 g/L, 0.1 g/L MgSO_4_·7 H_2_O. Under these optimized conditions, the predicted yield of MK-7 was 147.811 mg/L. Based on practical production considerations, the medium parameters were slightly adjusted to sucrose 20 g/L, glycerol 20.7 g/L, soybean peptone 47.3 g/L, yeast extract 4 g/L, and KH_2_PO_4_ 1.9 g/L, 0.1 g/L MgSO_4_·7 H_2_O. Validation experiments were conducted at the shake flask level under fermentation conditions of 40 °C and 220 rpm. The average MK-7 yield from three replicate experiments was 154.6 ± 1.32 mg/L, which closely matched the predicted value. These results demonstrate that the model predictions are stable and reliable, indicating their potential for guiding practical production applications.

## Discussion

Menaquinone-7 (MK-7), a vital lipophilic subtype of vitamin K_2_, has garnered significant attention for its broad applications in functional foods and dietary supplements (Ferland 2012). Among known MK-7-producing strains, *B.subtilis* stands out due to its well-characterized genetic background and generally recognized as safe (GRAS) status. In this study, systematic fermentation medium optimization was performed through single-factor experiments, Plackett-Burman design, steepest ascent experiments, and Box-Behnken response surface methodology. This approach achieved an MK-7 yield of 154.6 ± 1.32 mg/L, surpassing previously reported values under comparable optimization strategies(Wu and Ahn 2018; Li et al. 2023). While this yield remains lower than those of genetically modified hyper-producing strains (Chen et al. 2020; Sun et al. 2024), the key contribution of this work lies in demonstrating the feasibility of targeted genetic modifications within a simplified genomic context, thereby circumventing the genetic instability often associated with complex engineered strains. Such an approach offers greater potential for industrial scalability. Future research should integrate multi-omics analyses (e.g., metabolomics, transcriptomics) to elucidate carbon flux partitioning mechanisms and employ modular metabolic engineering to optimize key targets, advancing yield breakthroughs while maintaining genetic stability.

In industrial production, the adoption of low-cost culture media represents an effective strategy to reduce fermentation costs. For instance, Wu et al.(Wu et al. 2019) optimized the culture medium for *B.subtilis* WR350 through single-factor and orthogonal experiments, resulting in a reduction of fibrinolytic enzyme production costs to 23.35% of the initial level. Similarly, Cahyati et al.(Cahyati et al. 2021)successfully enhanced the yield of alkaline thermophilic xylanase by substituting pure glycerol with inexpensive technical glycerol (crude glycerol) and replacing peptone and yeast extract with soybean hydrolysate and rice bran. Building upon these strategies, the present study selected glycerol and sucrose as the optimized combined carbon sources: Glycerol, serving as the primary carbon source, not only offers cost advantages as a by-product of the biodiesel industry but also provides critical precursors for MK-7 biosynthesis. Studies have demonstrated that crude glycerol exhibits comparable fermentation efficacy to pure glycerol, thereby enabling its direct utilization to further reduce costs(El Kantar and Koubaa 2022). Sucrose, as an auxiliary carbon source, can be rapidly utilized during the cell growth phase to promote biomass accumulation while coordinating metabolic demands during the product synthesis phase, thereby enhancing overall productivity. Through the rational combination of such low-cost carbon sources, this research achieves cost reduction while maintaining production efficiency, laying a foundation for industrial scale-up.

Nitrogen sources serve as essential nutrients and regulatory factors for microbial growth and metabolite synthesis, and their cost-effectiveness directly influences the industrial feasibility of fermentation processes. Although high concentrations of soybean peptone (47.3 g/L) and yeast extract (4 g/L) in current media provide high-quality organic nitrogen, their elevated costs may hinder economic viability in large-scale production. To address this challenge, low-cost alternatives such as corn steep liquor (CSL), soybean meal hydrolysate, and ammonia water could be considered for partial substitution. CSL, a byproduct of the starch industry, is not only cost-effective but also rich in amino acids, organic acids, vitamins, and trace elements, making it a favorable nitrogen supplement for microbial fermentatio. It has been successfully employed in various fermentation systems(He et al. 2020). For instance, Tan et al. (Tan et al. 2016)demonstrated that CSL could effectively replace yeast extract in the high-efficiency cultivation of Actinobacillus succinogenes for succinic acid production. Similarly, Zeng et al. (Zeng et al. 2018) reduced the production cost of γ-polyglutamic acid by nearly 40% using a combination of CSL powder and cassava starch. Additionally, soybean meal hydrolysate, obtained via enzymatic or acidic hydrolysis, releases free amino acids, reducing metabolic burden while improving nitrogen utilization efficiency. Ammonia water, as an inorganic nitrogen source, can be directly assimilated into nitrogen metabolism, further decreasing reliance on organic nitrogen sources. To systematically enhance nitrogen utilization efficiency, future research could integrate metabolic engineering strategies, such as overexpressing nitrogen transporter proteins or disrupting nitrogen catabolite repression regulators, to develop high-performance nitrogen-assimilating strains. This combined approach—optimizing mixed nitrogen sources and employing strain engineering—holds significant potential to reduce fermentation costs while maintaining production efficiency, thereby offering a more competitive solution for industrial-scale applications.

Although shake flask optimization identified key medium components for MK-7 production, industrial-scale fermentation necessitates fed-batch bioreactors to overcome the inherent limitations of shake flask cultivation. The shake flask environment lacks precise control over critical parameters such as pH, dissolved oxygen (DO), and temperature, and cannot replicate the dynamic substrate feeding and metabolic regulation processes in bioreactors(Ganeshan et al. 2021). Moreover, single-batch feeding in shake flasks often leads to rapid substrate depletion or catabolite repression, thereby inhibiting MK-7 biosynthesis. In contrast, industrial-scale MK-7 production typically employs fed-batch bioreactors, where controlled substrate feeding and DO optimization enable higher cell density and product titers(Bisgaard et al. 2022). The optimized medium components (e.g., glycerol) identified in this study provide a critical foundation for designing a fed-batch strategy. To bridge the gap between shake flask optimization and industrial-scale fermentation, further refinement of feeding strategies and environmental control is essential.For instance, dynamic glycerol feeding can enhance MK-7 biosynthesis in cell membranes while preventing precursor accumulation-induced toxicity or wastage. Integrating substrate feeding with real-time feedback control of pH and DO can mitigate metabolic stress and maintain a stable cultivation environment. Additionally, scaling up the process to 1–5 L bioreactors under simulated industrial conditions will generate reliable data for further optimization and scale-up. Ultimately, transitioning from shake flasks to industrial bioreactors is not a simple medium transfer but a systematic integration of feeding strategies, environmental control, and scale-up logic. By combining the optimized medium with a controlled fed-batch fermentation strategy, the current shake flask findings can be translated into a scalable industrial process, thereby improving MK-7 production efficiency and economic feasibility.

Our research demonstrates that *B.subtilis*-based fermentation can achieve high MK-7 titers while maintaining industrial feasibility. Future studies should further integrate low-cost medium design, modular metabolic engineering, and scale-up process optimization to improve economic viability and facilitate commercialization.

## Conclusion

In this study, the fermentation process for MK-7 production by BS-Δ*ackA* was optimized with a focus on maximizing MK-7 yield. Initial single-factor optimization experiments were conducted using glycerol, sucrose, soybean peptone, yeast extract, KH_2_PO_4_, and MgSO_4_·7 H_2_O as variables. Through Plackett-Burman design experiments, the three factors exerting the most significant influence on MK-7 yield were identified as soybean peptone, glycerol, and KH_2_PO_4_. Subsequently, the steepest ascent experiment was employed to determine the optimal levels of these factors, which were then fine-tuned using the Box-Behnken response surface methodology. The optimized fermentation conditions were determined to be: sucrose 20 g/L, glycerol 20.65 g/L, soybean peptone 47.38 g/L, yeast extract 4 g/L, and KH_2_PO_4_ 1.88 g/L, 0.1 g/L MgSO_4_·7 H_2_O. Under these conditions, the MK-7 yield reached 147.811 mg/L. To ensure practical applicability, the medium composition was slightly adjusted to 20 g/L sucrose, 20.7 g/L glycerol, 47.3 g/L soybean peptone, 4 g/L yeast extract, and 1.9 g/L KH_2_PO_4_, 0.1 g/L MgSO_4_·7 H_2_O. Validation experiments conducted under these adjusted conditions yielded an average MK-7 production of 154.6 ± 1.32 mg/L in triplicate fermentations, which closely aligned with the predicted value. These results demonstrate the stability and reliability of the model predictions, confirming their suitability for guiding industrial-scale production. This study provides a robust theoretical foundation and technical framework for further optimization of the fermentation process for MK-7 production by *B.subtilis*, as well as its potential industrial applications.

## Data Availability

The datasets generated during and/or analysed during the current study are available from the corresponding author on reasonable request.
